# Determination of optimal time for reading of rapid urease test diagnosis of *Helicobacter pylori*

**Published:** 2020

**Authors:** Alireza Eslaminejad, Sayed Mehran Marashian, Maryam Aboutorabi, Makan Sadr, Shahram Agah

**Affiliations:** 1 *Chronic Respiratory Diseases Research Center, National Research Institute of Tuberculosis and Lung Disease, Masih Daneshvari Hospital, Shahid Beheshti University of Medical Sciences, Tehran, Iran*; 2 *Virology Research Center, National Research Institute of Tuberculosis and Lung Disease, Masih Daneshvari Hospital, Shahid Beheshti University of Medical Sciences, Tehran, Iran*; 3 *Colorectal Research Center, Iran University of Medical Sciences, Tehran, Iran*

**Keywords:** Helicobacter pylori, Rapid urease test (RUT), Diagnostic value, Pathophysiology

## Abstract

**Aim:**

The study aimed to find the best reading time for the best accuracy of RUT in optimal time to obtain faster results with lower false rates and consequently save time in commencing treatment of peptic ulcers.

**Background:**

Rapid urease test is well known to be an accurate test for *H.pylori* detection in tissue biopsies.

**Methods:**

Patients with GI problems referring to a university hospital in Tehran who underwent endoscopy and biopsy were entered in the project and three samples of mucosal tissue were captured from the lesser curvature, the antrum and the body of stomach.

**Results:**

We found 39.6% sensitivity and 95% specificity for the named test in the first 5 minutes as well as PPV = 95.5% and NPV = 37.3% while the accuracy was 54.79%. Except for the specificity which was constantly 95% in all RUT reading times, other diagnostic parameters increased as time went on. The PPV was also higher than 97% after 10 minutes. The highest values of sensitivity, specificity, PPV, NPV and accuracy were achieved after 12 hours including 88.7%, 95%, 97.9%, 76% and 90.41%, respectively.

**Conclusion:**

To conclude, it seems that there are many different ideas with respect to the rapid urease test in *H.pylori* detection. However, the current study recommends reading the test optimally after 12 hours but it is suggested more multidisciplinary studies with bigger sample size be carried out to obtain better and more reliable results to be able to generalize in this regard.

## Introduction

*H.pylori* infection affects more than half the world’s population and more adults in developing countries are infected. *H.pylori* infection is the main cause of dyspepsia in 10% of people and is also the main correlated factor in duodenal and gastric ulcers in 95% and 70% of cases, respectively (1, 2). Rapid urease test (RUT) is an indirect test for diagnosis of *H.pylori* according to the presence of urease in the gastric mucosa. RUT is a common rapid cheap and simple diagnostic test and its important advantage over serology tests is in its detection of active infectious agents (3). 

Diagnosing *H.pylori* infection has been made based on biopsies and pathology for a long time and this technique is known as the golden standard test to date. During recent decades, physicians have decided to use techniques like genetic studies as well as urease tests as they are more applicable, faster and probably more accurate and cost-effective.

Rapid urease test (RUT) includes a high urea containing media with an indicator which is sensitive to PH and changes color at different PH rates. A study by Foroutan et al. in 2010 identified RUT 98.57% sensitive, 99.29% specific and accurate in 99.04% of stomach biopsies (4). Regarding the importance of diagnosis time and treatment of peptic ulcers, RUT is a crucial, accurate and fast test. This test needs 24 hours before reading but different reading times have their own different sensitivity and specificity and we may lessen the time to get acceptable results when positive. In 1996 it was disclosed that *H.pylori* population in the sample tissues strongly affects the speed of obtaining positive results. For instance, a RUT positive result in 20 minutes needs a population of between 3*10^2^ and 3*10^3^ microorganisms (5). Laine et al. raised the importance of multiple tissue biopsies in decreasing the time taken to read RUT results although 40% false negative rate was seen (5).

The current diagnostic study headed to find the best reading time for the best accuracy, sensitivity and specificityof RUT as well as the optimal time of positivity to get faster results with lower false rates and save time in the commencement of treatment of peptic ulcers. 

## Methods

Through an analytical cross-sectional prospective study, patients with GI problems referring to a university hospital in Tehran who underwent endoscopy and stomach biopsy were enrolled in the current research project. People with active upper GI bleeding, with a history of gastrectomy and hypotension (SBP<90 mmHg) were excluded from this study. Patients who had taken antibioticstwo weeks before the study, including bismuth derivatives and proton pump inhibitors left the study as well. 

**Endoscopy and biopsy: **Heretofore, histopathology was the diagnostic choice to distinguish gastritis and/or peptic ulcers due to *Helicobacter pylori (H.pylori)*. To obtain optimal results, three samples of mucosal tissue from the stomach are routinely captured using large-cap forceps from the lesser curvature, the antrum and the body of stomach. Biopsy samples gatheredfrom the patients were transferred to the pathology department for H&E and Giemsa staining before a microscopic study. H&E stain and microscopy is an excellent test in big populations of *H.pylori* but the organism is not always abundant enough to achieve perfect microscopic assessment. 

**Rapid Urease Test (RUT): **RUT is a kind of urease test in which hydrolyses of urea products into ammonia and CO^2^ is performed as follows: The produced ammonia alkalizes samples containing medium to force the PH to change the color of the existing indicator. 

(NH_2_)^2^ CO + 2 H_2_O → CO_2_ + H_2_O + 2NH_3_

This study used RUT agar kits by Shimanzim ®, Iran with phenol red as PH indicator. Positive results for *H.pylori* were considered when the color of the kits changed from light orange into pink/red in 24 hours. Color changes were recorded in 5, 10, 20 and 60 minutes and then after 2, 6, 12 and 24 hours. 

**Statistics: **Data entered in the SPSS and EPI info16 and frequency and central tendency indices as well as the assessments for accuracy, sensitivity and specificity of RUT were compared with the golden standard pathology. The power of the study was headed to be 0.8 beside type one error (α = 0.05) and the confidence interval (CI = 95%). 

**Ethics: **The current study recruited candidate patients for diagnostic endoscopy regarding the medical indications and physician decision by census. There was no obligation to do the tests with no indication. The costs of the diagnostic procedures were covered by the project and the participants with positive results for *H.pylori* infection were prescribed for treatment. All the participants’ information was secured by the principle investigator. 

## Results

In the present study 264 patients with endoscopy and biopsy results were enrolled and among them 118 had used antibiotics in recent weeks and were excluded from the study. Finally, 146 patients including 86 (58.9%) females and 60 (41.1%) males participated in this project. The mean age and standard deviation were reported 45.36 ± 17.41 years with 25% of patients who were over 59.25 years. 

Nausea and vomiting were reported by 30 subjects (20.5%) while 64 (43.8%) had abdominal pain when fasting but 60 (41.1%) complained of stomachache after eating. Flatulence (42.5%), heartburn (26%), haematochesia (2.7%), melena (8.2%), anemia (5.5%) and occult blood (2.7%) were the other findings in the studied population. A history of upper GI bleeding was raised in 14 (9.6%) and among them, 2 (1.4%) had had it in the recent month. Six patients (4.1%) in recent 1-6 months, 2 (1.4%) in previous 6-12 months and 2 (1.4%) in the last year had Upper GI bleeding.

**Figure 1:**
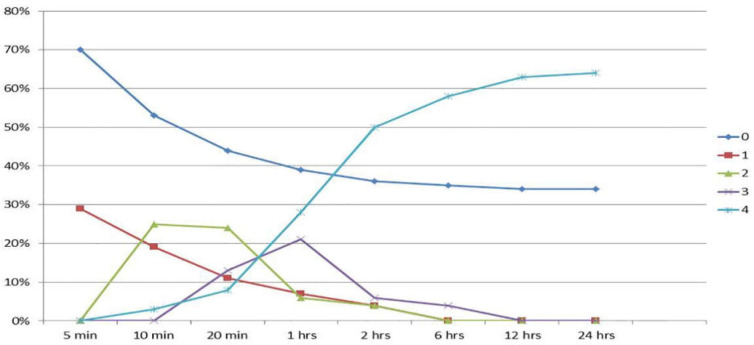
Trend of RUT results during 24 hours: 0=no changes in the color of the specimen; 1=+1: Shows a color change in only a part of the tissue; 2=+2: Color change in all the tissue with 1 mm margin around it; 3=+3: Between +2 and +4; 4=+4: Complete color change in the whole solution

Concerning RUT, 102 (69.9%) showed negative results after 5 minutes compared to 44 (30.1%) positive casesof *H.pylori*. The RUT results were followed for 24 hours in the participants. Table 1 and figure 1 illustrate the findings in this regard in more detail as well as the trend of each stage of the test throughout the study.

**Table1:** Frequency of RUT and pathology results

		Pathology		Total(%)
		Yes (%)	No(%)	
RUT5	Yes	42(95.5)	2(4.5)	44(100)
	No	64(62.7)	38(37.3)	102(100)
RUT10	Yes	66(97.1)	2(2.9)	68(100)
	No	40(51.3)	38(48.7)	78(100)
RUT20	Yes	78(97.5)	2(2.5)	80(100)
	No	26(40.6)	38(59.4)	64(100)
RUT1hrs	Yes	86(97.7)	2(2.3)	88(100)
	No	18(32.1)	38(67.9)	56(100)
RUT2hrs	Yes	90(97.8)	2(2.2)	92(100)
	No	14(26.9)	38(73.1)	52(100)
RUT6hrs	Yes	92(97.9)	2(2.1)	94(100)
	No	12(24)	38(76)	50(100)
RUT12hrs	Yes	94(97.9)	2(2.1)	96(100)
	No	12(24)	38(76)	50(100)
RUT24hrs	Yes	94(97.9)	2(2.1)	96(100)
	No	12(24)	38(76)	50(100)

The golden standard test in this work was histopathology of stomach biopsies as discussed in the methodology section. Overall, 94 patients (64.4%) presented with *H.pylori* infection in the body of their stomach based on the above test but the rest were negative. The antrum seemed to be more infected than the body as reported by positive pathologic results for 98 (67.1%). So, the positivity of pathology for infection was 106 (72.6%) regardless of the location of it. 

A strong linear correlation was found between RUT results and pathology results in biopsies from body part of the stomach at different times of the test from 5 minutes to 24 hours (P value < 0.001). A similar correlation was shown in a comparison between antrum pathologic studies and RUT (P value < 0.001) with rather higher coefficients. 

Interestingly, body infection was obviously correlated with antrum infection in pathology (P value < 0.001; r = 0.639). People with positive pathology results for *H.pylori* infection were overall around 10 years older than the people with negative results (48.47 ± 16.46 *vs.*37.1 ± 17.33 years) (P value < 0.001). City of settlement, sub nationality and education were not matters of significant difference in pathology. 

In order to find the sensitivity, specificity and predictive values for RUT, we compared the findings with the golden diagnostic test and found 39.6% sensitivity and 95% specificity for the named test in the first 5 minutes as well as PPV = 95.5% and NPV = 37.3% while the accuracy was 54.79% (table 2). Except for the specificity which was constantly 95% in all RUT reading times, other diagnostic parameters were 

**Table2:** Sensitivity, specificity, PPV, NPV and accuracy of RUT in different reading times

RUT	Sensitivity (%) CI 95%	Specificity (%) CI 95%	PPV (%) CI 95%	NPV (%) CI 95%	Accuracy CI 95%
5 min	39.6 26.8-54	95 73.1-99.7	95.5 75.1-99.8	37.3 24.5-51.9	54.79
10 min	62.3 47.9-74.9	95 73.1-99.7	97.1 82.9-99.8	48.7 32.7-65	71.23
20 min	75 60.08-85.5	95 73.1-99.7	97.5 85.3-99.9	59.4 40.8-75.8	80.55
1 hour	82.7 69.2-91.3	95 73.1-99.7	97.7 86.5-99.9	67.9 47.6-83.4	86.11
2 hours	86.5 73.6-94	95 73.1-99.7	97.8 87-99.9	73.1 51.9-87.6	88.88
6 hours	88.5 75.9-95.2	95 73.1-99.7	97.9 87.3-99.9	76 54.5-89.8	90.27
12 hours	88.7 76.3-95.3	95 73.1-99.7	97.9 87.5-99.9	76 54.5-89.8	90.41
24 hours	88.7 76.3-95.3	95 73.1-99.7	97.9 87.5-99.9	76 54.5-89.8	90.41

growing as time went on. The PPV was also higher than 97% after 10 minutes and had the least changes as can be seen in table 4. Finally, the highest values of sensitivity, specificity, PPV, NPV and accuracy were achieved after 12 hours including 88.7%, 95%, 97.9%, 76% and 90.41%, respectively and the future results continued to be exactly the same.

In terms of more details in diagnostic value of RUT, we calculated positive and negative likelihood ratios for the 12^th^ hour of the test using the following equations:

$\mathrm{Likelihood ratio for positive results}=\frac{\mathrm{Sensitivity}}{\left(1-\mathrm{Specificity}\right)}=17.74$

$\mathrm{Likelihood ratio for negative results}=\frac{\left(1-\mathrm{Sensitivity}\right)}{\mathrm{Specificity}}=0.12$

The above likelihood ratio for RUT results means that its positivity can unremarkably increase the probability of gastritis and/or ulcers while negative results show a more reliable role of the test to rule out *H.pylori* active infection.

## Discussion

The current study showed the most effective values of the assessed diagnostic factors at the 12^th^ hour of RUT test and recommendsthat the best time to read RUT results is after 12 hours instead of 24 hours. We used three biopsy samples for each patient because we could not find the effect of size and number of biopsies on the rate and time of positive results in RUT similar to what happened in Laine’s study published in 1996. They found that two biopsy samples could make results positive 30 minutes earlier in 56% of cases compared with a single biopsy sample (5). They also found better sensitivity using two samples although 40% false negative cases occurred in earlier readings. Most of the false negative results in RUT may be due to taking antibiotics, PPIs and presence of intestinal metaplasia as Uotani clarified before (6). RUT, using urease, has superiorityover serologic tests because of detecting the active phase of infection and not latent infections (6). In any case, regarding the minimum number of organisms to make the results positive (10^5^), the quality and location of the samples are the most important parts of the test. For instance, atrophic lesions in the stomach cannot be good sampling sites because of few organisms and by pushing the results to false negativity more than even a single sample from duodenal non-atrophic lesions which perfectly provide enough of a *H.pylori* population to make RUT sensitivity and specificity more optimal and consequently more reliable (6). However, this minimum number of organisms is not significantly important because the organisms usually exceed 105 and this would provide high sensitivity in easier steps of the test as the current study showed (7-11).

A study by Levin et al. in 2005 showed 100% specificity of the test disregarding the time of reading but around 31% of early negative results changed into positive in RUT_24_ and 9.8% of the whole cases were positive in results in histopathology but reported negative by both RUT_0_ and RUT_24_ (12). They believed that these false negative cases were due to imperfect sampling, in that PPIs or other medications could not alter the sensitivity and specificity of RUT at all. On the contrary, a study in 2000 in Greece distinguished that RUT, with its own diagnostic values, could not discover all the positive cases of *H.pylori* infection reported by histopathology, especially during the first 24 hours (13). Similarly, Talebi et al. in a letter to the Saudi journal of gastroenterology in 2011 asked physicians not to rely on RUT as a sensitive test of detection for *H.pylori* but on the contrary Foroutan et al. who declared the test as being a reliable test in 2010 because of finding 30% false negative results by the test (4, 14).

Peptic ulcers are causative factors of dyspepsia in 10% of cases and 95% of duodenal and 70% of gastric ulcers are mainly due to *H.pylori* (2). So, controlling the infection is a crucial treatment for curing patients. In this regard, accurate fast detection plays the most predominant role and RUT is usually faster than histopathologic studies, especially if the results are reliable enough after 12 hours as our study has shown. Unlike histopathology, RUT does not need any specialty or subspecialty nor any complex process to make it cheaper. The other advantage is theability of carrying out RUT using portable kits with no necessary equipment , possible in the most remotest of areas. 

The prevalence of the disease is important when likelihood ratios for positive and negative RUT results are concerned. There are several studies on the worldwide prevalence of *H.pylori* infection; some of which have been conducted in Iran showing 90% infection rate among adults and involvement of children in more than 50% before the age of 15 (15, 16, 17).

 Among hospital referrals of dyspepsia, a range of 60-80% is usually reported for the prevalence of *H.pylori* infection by histopathology (18-20). Looking at the mentioned rates, likelihood ratios would be applied as follows (Probability _Pre_):

For positive RUT results: 

$\mathrm{Odds ratio Pre}=\frac{\mathrm{Probability Pre}}{\left(1-\mathrm{Probability Pre}\right)}=\frac{0.7}{1-0.7}=2.33$

Odds Post = Odds Pre × LR + = 2.33×17.74 = 41.33

$\mathrm{Probability Post}=\frac{\mathrm{Odds Post}}{1+\mathrm{Odds Post}}=\frac{41.33}{42.33}=0.98$

The odds ratio here shows the actual effect of LR on the probability of *H.pylori* infection among patients with dyspepsia. Regarding the values of LR+, when a patient with dyspepsia refers to us and the RUT is positive in 12 hours, it means our patient is suffering from *H.pylori* active infection with a 98% chance. In other words we can increase the chance of infection diagnosis from 70% at the beginning to 98% by a simple 12-hour test in a hospital setting. For negative results the probability _Post_ was obtained at 22% showing that the first 70% chance of *H.pylori* changes into 22% if RUT is negative in 12 hours. So, RUT positive results can draw up the diagnosis to 28% whilst 48% in negative results. With these diagnostic values, is it not feasible that we can rely on RUT and reduce the use of pathologic studies in this regard, especially in areas where we have a lack of equipment, personnel, and finance? 

To conclude, it seems that there are many different ideas with respect to rapid urease test in *H.pylori* detection. However, the current study recommends reading the test optimally after 12 hours and we also advise more multidisciplinary studies be carried out with a bigger sample size to obtain better and more reliable results to be able to generalize in this regard.

## Conflict of interests

The authors declare that they have no conflict of interest.
